# Compounds purified from edible fungi fight against chronic inflammation through oxidative stress regulation

**DOI:** 10.3389/fphar.2022.974794

**Published:** 2022-09-09

**Authors:** Yidan Xia, Dongxu Wang, Jiaqi Li, Minqi Chen, Duo Wang, Ziping Jiang, Bin Liu

**Affiliations:** ^1^ Department of Hand and Foot Surgery, The First Hospital of Jilin University, Changchun, China; ^2^ Laboratory Animal Center, College of Animal Science, Jilin University, Changchun, China

**Keywords:** chronic diseases, natural compounds, edible fungi, antioxidants, molecular mechanisms

## Abstract

Chronic inflammation is associated with various chronic diseases, including cardiovascular disease, neurodegenerative disease, and cancer, which severely affect the health and quality of life of people. Oxidative stress induced by unbalanced production and elimination of reactive oxygen species (ROS) is one of the essential risk factors for chronic inflammation. Recent studies, including the studies of mushrooms, which have received considerable attention, report that the antioxidant effects of natural compounds have more advantages than synthetic antioxidants. Mushrooms have been consumed by humans as precious nourishment for 3,000 years, and so far, more than 350 types have been identified in China. Mushrooms are rich in polysaccharides, peptides, polyphenols, alkaloids, and terpenoids and are associated with several healthy biological functions, especially antioxidant properties. As such, the extracts purified from mushrooms could activate the expression of antioxidant enzymes through the Keap1/Nrf2/ARE pathway to neutralize excessive ROS and inhibit ROS-induced chronic inflammation through the NF-κB pathway. Recently, the antioxidant properties of mushrooms have been successfully applied to treating cardiovascular disease (CAD), neurodegenerative diseases, diabetes mellitus, and cancer. The present review summarizes the antioxidant properties and the mechanism of compounds purified from mushrooms, emphasizing the oxidative stress regulation of mushrooms to fight against chronic inflammation.

## Introduction

Mushrooms have been extensively cultivated in China using artificial techniques due to their high yield, desirable taste, and widespread consumption and application as a medicinal resource (Huang and Nie, 2015). Recently, various compounds have been isolated from mushrooms, such as polysaccharides, alkaloids, peptides, terpenoids, and polyphenols (Homer and Sperry, 2017; Zhou et al., 2020; Kuang et al., 2021; Leong et al., 2021; Zhang et al., 2021). Mushroom extracts can resist free radicals, reduce the activities of pro-inflammatory factors, and relieve chronic inflammation, which are valuable natural antioxidants with the advantages of safety, nontoxic, and easy to obtain (Hu et al., 2021). Some studies have also demonstrated the therapeutic potential of mushroom extract for cardiovascular disease (CAD), neurodegenerative diseases, and cancer (Klupp et al., 2015; Jin et al., 2016; Chun et al., 2021). This review discusses the mechanisms underlying the effect of oxidative stress on chronic inflammation and summarizes the antioxidant properties of compounds purified from mushrooms. The anti-inflammatory effects of these compounds on CAD, neurodegenerative diseases, diabetes mellitus, and cancer provide potential treatment measures for chronic inflammation caused by oxidative stress.

## Chronic inflammation and oxidative stress

### Predisposing factors of oxidative stress

The predisposing factors, including diet, exercise, chemicals, radiation, and drugs, can increase reactive oxygen species (ROS) production and disrupt the antioxidant system, inducing oxidative stress (Klaunig et al., 2011; Quindry et al., 2016; Tan et al., 2018; Tan and Norhaizan, 2019). Similarly, eating habits, including refined carbohydrates, high-fat, and high-animal protein diets, can increase oxidative stress through the nuclear factor-kappa B (NF-κB) signaling pathways (Tan et al., 2018). High sugar intake can produce advanced glycation end products to promote oxidation and activate the protein phosphatase 2 A and NF-κB pathways, inducing oxidative stress in mitochondria (Man et al., 2020). Regular and moderate exercise has also been proven to inhibit ROS production, while high intensity and long-term exercise might induce oxidative stress in the skeletal muscle cells, causing muscle contractile dysfunction (Powers and Jackson, 2008; Thirupathi et al., 2020). A previous study has reported that the exposure of cells to metals and metal oxide nanoparticles induces oxidative stress, thereby damaging the deoxyribonucleic acids (DNAs), proteins, and lipids (Tee et al., 2016). For instance, aluminum accumulation could impair the oxidative function of mitochondria, especially in the brain, which is explicitly sensitive to oxidative stress and is one of the causes of neurodegenerative diseases (Kumar and Gill, 2014). Similarly, continuous exposure to ionizing radiation induces excessive production of hydroxyl radicals through water oxidation or secondary partially ROS formation (Klaunig et al., 2011). Drugs, including gentamicin and bleomycin, could produce free radicals during degeneration and metabolism (Pizzino et al., 2017). Additionally, the potential predisposing factors, including unhealthy living habits, exposure to harmful substances, and certain drugs, could induce oxidative stress and damage the cellular components through different mechanisms.

### Mechanism of oxidative stress

**FIGURE 1:**
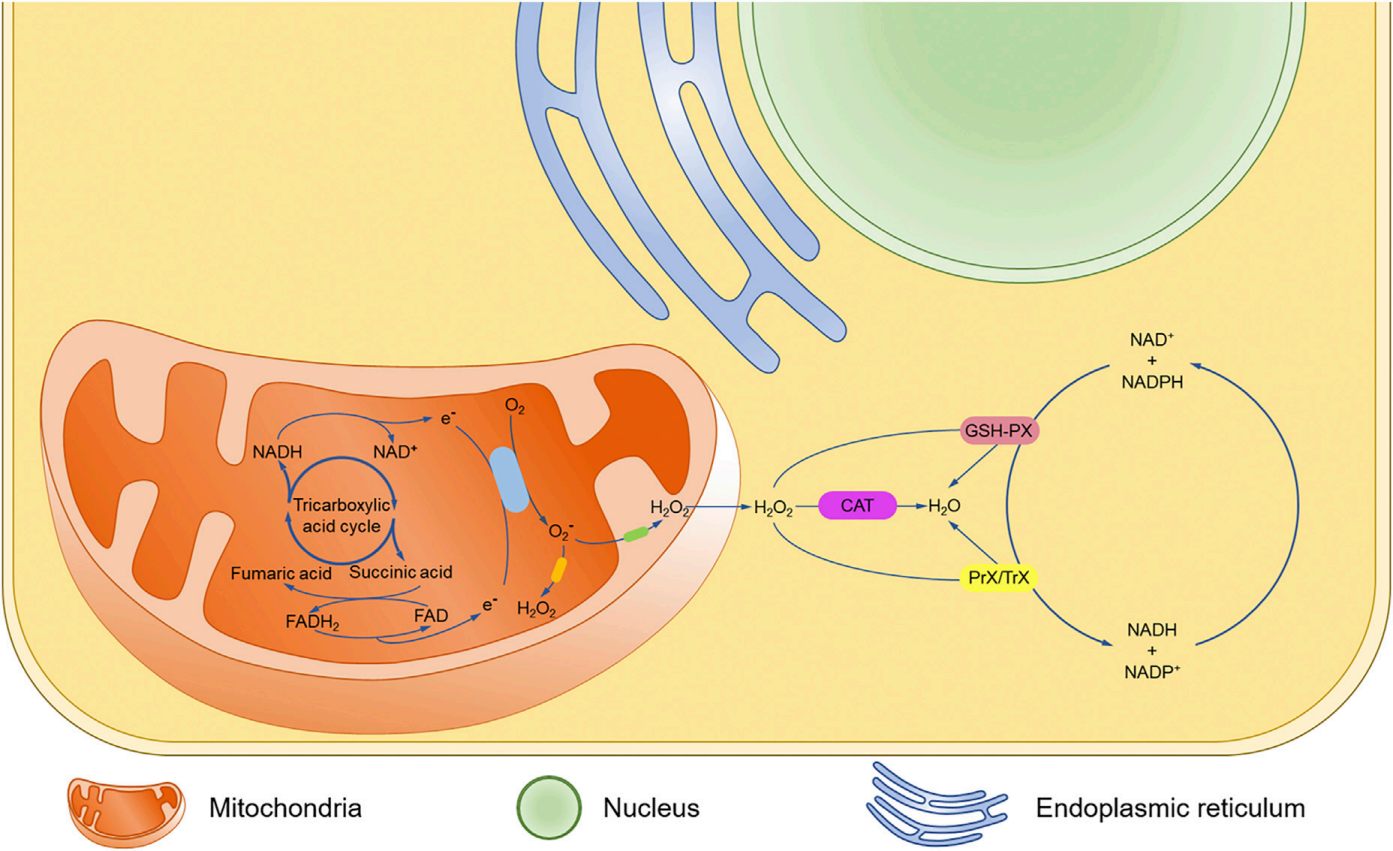
Mechanism of oxidative stress.

In mitochondria, most oxygen is utilized for aerobic respiration and energy release, with ROS being produced as a byproduct (Cadenas and Davies, 2000). Mitochondria convert glucose to adenosine triphosphate (ATP) through the tricarboxylic acid cycle and oxidative phosphorylation by using nicotinamide adenine dinucleotide (NADH) and reduced flavin adenosine dinucleotide (FADH2) produced in the cytoplasmic matrix (Ryoo and Kwak, 2018). In the oxidative phosphorylation process, NADH and FADH2 efficiently reduce oxygen and release energy through the electron transport chain (ETC) (Bhatti et al., 2017). However, a small quantity of oxygen is still reduced prematurely by electrons, producing superoxide anion (O_2_^−^), a relatively stable intermediate, which can generate hydrogen peroxide (H_2_O_2_) by disproportionation reaction, or hydroxyl radicals (OH) by the Haber-Weiss and Fenton reaction (Figure 1) (Sinha et al., 2013).

Under physiological conditions, ROS can be removed by the antioxidant enzymes, including superoxide dismutase (SOD), catalase (CAT), glutathione peroxidase (GSH-PX), and peroxiredoxin/thioredoxin (Prx/Trx) (Griendling et al., 2021). SOD consists of manganese superoxide dismutase (MnSOD) located in the mitochondrial matrix and cuprum/zinc superoxide dismutase (Cu/ZnSOD) in the membrane gap, which can dismutate the O_2_—produced by ETC into H_2_O_2_ (Peoples et al., 2019). The GSH-PX and Prx/Trx systems of the cytoplasmic matrix could reduce H_2_O_2_, while nicotinamide adenine dinucleotide phosphate (NADPH) maintains the reducing power of these two antioxidant systems (Nolfi-Donegan et al., 2020). Meanwhile, NADH/NADPH oxidase serves as an intracellular ROS source, especially in the vascular tissues and cardiomyocytes (Elahi et al., 2009). The persistence of ROS damages the somatic cells, including the cardiac muscle cells and nerve cells, while the antioxidant properties of the compounds in mushrooms neutralize the ROS (Chun et al., 2021).

### Mechanism of oxidative stress in chronic inflammation

**FIGURE 2:**
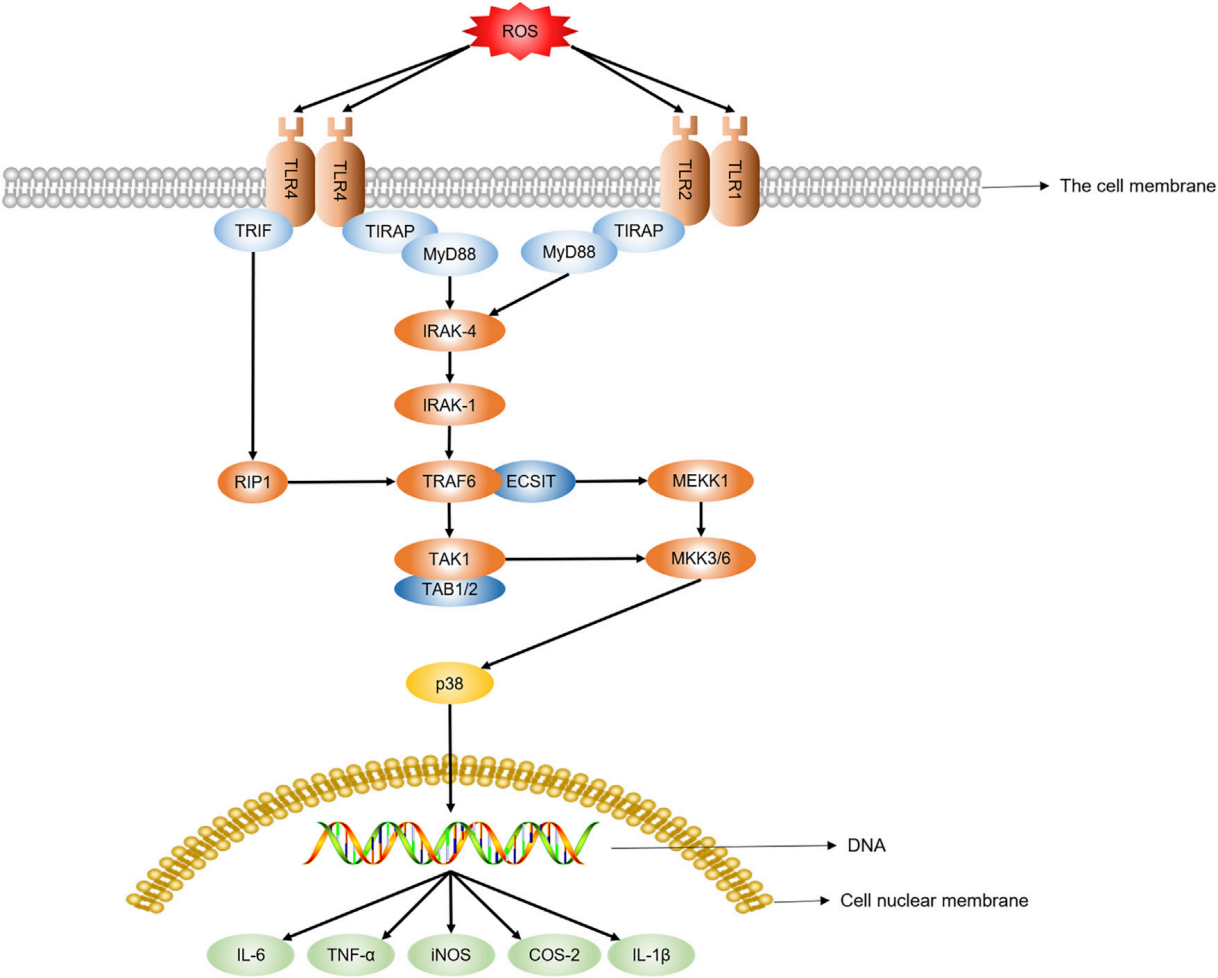
ROS promote pro-inflammatory factors release through the NF-KB pathway.

The persistence of inflammatory factors causing tissue damage, such as trauma, chemical erosion, microbial infection, and autoimmune reaction, significantly contributes to chronic inflammation (Medzhitov, 2008; Leuti et al., 2020). When inflammation occurs, oxygen uptake by the white blood cells and mast cells in the inflammatory area increases, leading to “respiratory burst,” which enhances ROS production and release (Reuter et al., 2010). It is reported that low density lipoprotein (LDL) can be oxidized by ROS and phagocytosed by macrophages, thereby improving the release of pro-inflammatory factors and inducing inflammatory responses mediated by the NF-κB signal pathway (Zuo et al., 2019). The expression of phagocytic NADPH oxidase relying on NF-κB could produce ROS and increase the bactericidal activity of macrophages through the Toll-like receptors, including Toll-like receptor 1 (TLR1), TLR2, and TLR4 (West et al., 2011). However, excessive intracellular ROS stimulate the expression of NF-κB by activating the p38 signaling pathway and regulating the liberation of pro-inflammatory factors, including tumor necrosis factor-α (TNF-α), inducible nitric oxide synthase (iNOS), and cyclooxygenase-2 (COX-2) (Xi et al., 2020). Additionally, ROS could stimulate the expression of TLR4 and trigger an inflammatory response in the lungs by activating the NF-κB pathway (Mishra et al., 2018). Oxidative stress can induce chronic inflammation, while the inflammatory response can improve ROS release, primarily mediated through the NF-κB signal pathway (Figure 2).

## Antioxidant effects and mechanisms of compounds purified from edible fungi

### Extraction of compounds from edible fungi

Mushrooms are rich in polysaccharides, peptides, polyphenols, and other compounds beneficial to human health. Hence, mushrooms have been widely recognized as functional food resources and are being used for developing drugs and nutrients (Alves et al., 2012). In recent years, mushroom-derived polysaccharides have gained considerable attention due to their antioxidant, antibacterial, anti-tumor, and immunomodulatory effects (Wang et al., 2017). Most of the polysaccharides from mushrooms are water-soluble and can be extracted frequently by water extraction using heating, ultrasound, and microwave techniques (Leong et al., 2021). According to modern technology, water extraction has some disadvantages, such as long extraction time, low efficiency, and risk of biopolymer degradation. As such, the subcritical pressurized hot water extraction technology and deep eutectic solvent extraction technology have emerged as potential approaches to achieving efficient water extraction (Rodrigues Barbosa et al., 2020). Extracting mushroom-derived bioactive peptides is mostly dependent on the proteolytic action of exogenous enzymes and microbial fermentation technology (Zhou et al., 2020). Traditionally, microwave-assisted and ultrasound-assisted methods were used to extract the phenolic compounds (Petrovic et al., 2014). Different extraction methods may influence the antioxidant effects of different compounds in mushrooms, thus helping to select the appropriate extraction method to inhibit chronic inflammation by eliminating ROS.

### Polysaccharide

**FIGURE 3:**
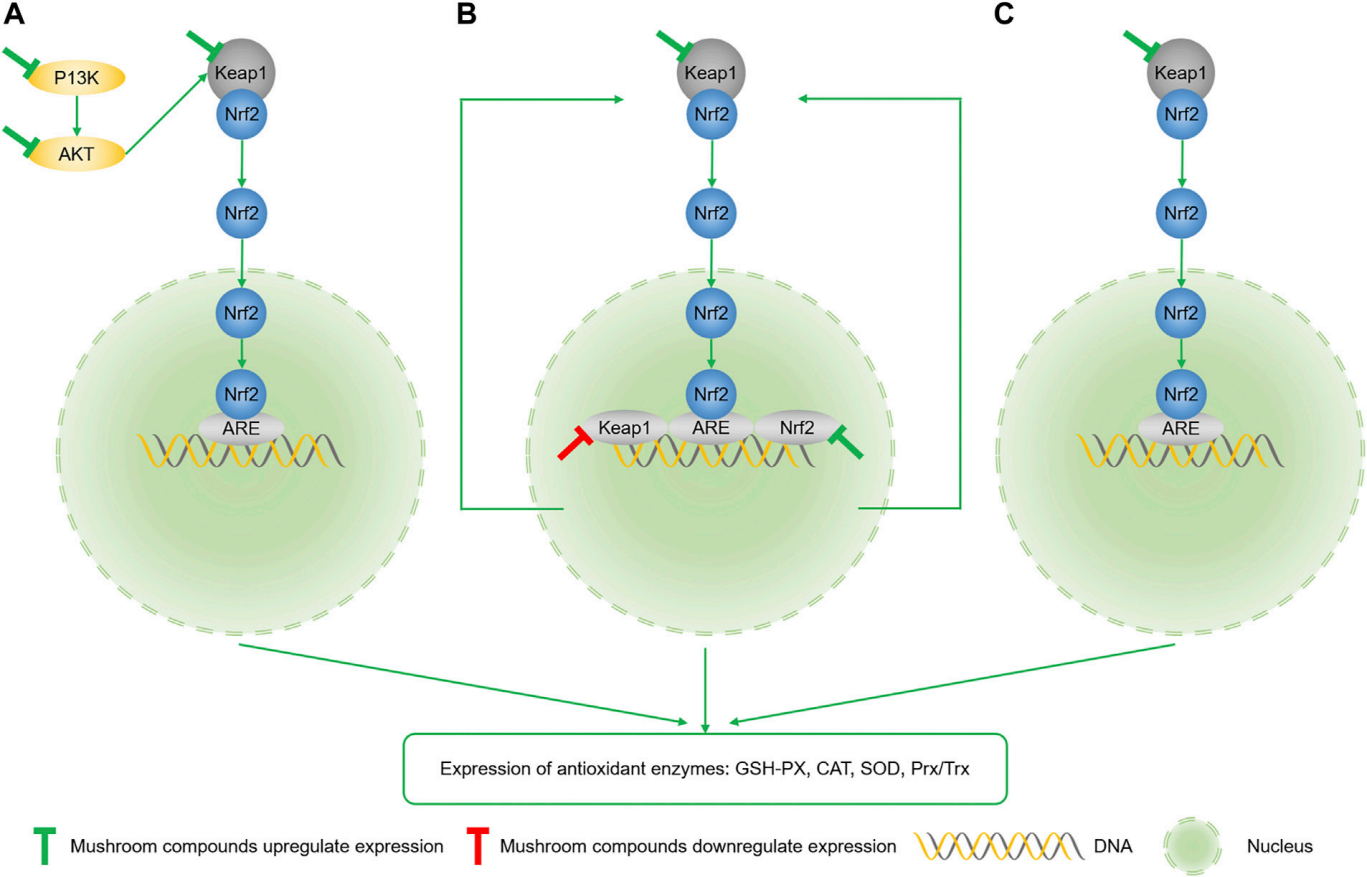
**(A)** Mushroom polysaccharides activate the Keap1/Nrf2/ARE pathway by upregulating the expression of P13K and AKT. **(B)** Mushroom peptides activate the Nrf2 pathway by downregulating the Keap1 gene and upregulating the Nrf2 gene expression. **(C)** Polyphenols directly promote the dissociation of Keap1 and Nrf2.

Polysaccharide, the most widely studied compound in mushrooms, is primarily composed of more than 10 monosaccharides linked with glycosidic bonds of polymeric sugar polymer carbohydrates (Supplementary Figure.S1) (Leong et al., 2021). The hydrogen ion on the sugar chain of polysaccharides can directly bind to OH and break down into harmless products (Lu et al., 2020). Polysaccharides can inhibit oxidative stress by improving the activities of antioxidant enzymes, including SOD, CAT, and GSH-PX, or chelate with metal ions, essential for ROS production (Huang et al., 2017). The antioxidant effect of polysaccharides is mainly mediated through the activation of the Keap1/Nrf2/ARE pathway to achieve the expression of downstream antioxidant enzymes (Figure 3A) (Chen et al., 2021). Nuclear factor erythroid-2 (Nrf2) is the major regulatory element of the antioxidant system *in vivo*, which can detach from the binding site of Kelch-like ECH-associated protein 1 (Keap1) in the cytoplasm after being stimulated and transferred to the nucleus to bind with the anti-oxidant response element (ARE) (Liao et al., 2019). Polysaccharides have been reported to regulate oxidative stress by upregulating the expression of phosphatidylinositol 3-kinase (P13K) and phosphorylation of protein kinase B (AKT) (Yun et al., 2020).

Poria cocos polysaccharide could eliminate O_2_—and OH, while carboxymethylated pachymaran could promote the expression of SOD to achieve an antioxidant effect (Li et al., 2019). Lepista nuda polysaccharides extracted through water extraction and alcohol precipitation method could chelate iron ions and scavenge O_2_—and 1,1-diphenyl-2-picrylhydrazyl free (DPPH) radicals and in a concentration-dependent manner (Shu et al., 2019). A water-soluble polysaccharide isolated from the alkaline extract of Entoloma lividoalbum could eliminate OH through the hydrogen atom donation ability of its hydroxyl group (Maity et al., 2014). In a previous study, the ultrasonic-assisted extraction of Flammulina velutipes polysaccharide showed stronger scavenging ability of DPPH, OH, and O_2_—than hot water extraction, with better inflammation inhibitory ability (Chen et al., 2019). For instance, the polysaccharide purified for the first time from the floral mushrooms cultivated in Huangshan could scavenge 79.46% DPPH free radical and 74.18% OH at 5 mg/ml (Wang et al., 2015). A new polysaccharide AAP-3-1 isolated from the fruit body of Auricularia auricula could inhibit ROS production, reduce malondialdehyde content, and increase the activities of SOD, GSH-PX, and CAT (Qian et al., 2020).

### Peptides

Peptides are composed of three or more amino acid molecules linked by peptide bonds, which can be easily absorbed by the intestine and pose excellent physiological characteristics than proteins (Erdmann et al., 2008). The aromatic amino acids and hydrophobic amino acid residues in the peptides could significantly increase the antioxidant activity, thereby inducing hydrogen atoms (Fontoura et al., 2019; Yang et al., 2020). Peptides can activate the Nrf2 pathway by downregulating the Keap1 gene and upregulating the Nrf2 gene expression to achieve antioxidant effects (Figure 3B) (Liu et al., 2021). Mushrooms are the primary sources of natural active peptides with significant potency, high tissue affinity, low toxicity, and high stability (Zhou et al., 2020). An analysis of the GSH levels in various mushroom species showed that the GSH content of Maitake was higher than most fruits and vegetables, indicating that mushrooms could be an indispensable source of GSH in the daily diet (Kalaras et al., 2017). In a previous study, Matsutake derived peptide WFNNAGP significantly inhibited glucan sulfate-induced oxidative stress in mice by scavenging OH and promoting the SOD activity, thereby preventing colon inflammation (Li et al., 2021a). Low molecular weight peptides decomposed from the protein hydrolysates of *Agaricus bisporus* by enzymatic processes are abundant in negatively charged amino acids, which can be applied to neutralize free radicals to resist oxidative stress (Kimatu et al., 2017).

### Polyphenol

Polyphenols consisting of at least one aromatic ring with hydroxyl functional groups, including flavonoids, phenolic acids, stilbenes, and lignans, are the natural antioxidants in plant foods (Kozarski et al., 2015). Polyphenols can provide electrons or hydrogen atoms to neutralize free radicals or chelate with metal ions to reduce the rate of Fenton reaction (Figure 3C) (Cheng et al., 2017). *Agaricus brasiliensis* mushroom containing phenolic compounds, such as gallic acid, serum acid, and pyrogallol, could inhibit ROS production by tert-butyl hydrogen peroxide stimulated macrophages (Navegantes-Lima et al., 2020). A novel polyphenol isolated from Phellinus linteus hispolon and its derivatives has been reported to have a strong free radical scavenging ability (Sarfraz et al., 2020). In a previous study, the release of phenolic compounds in agaricus bisporus, cantharellus cibarius, and lentinula edodes was determined by the simulating human gastrointestinal digestion method. The results indicated that lentinus edodes released the most phenolic acids, confirming that the phenolic compounds in lentinus edodes might easily improve the antioxidant capacity of the human body through consumption (Kala et al., 2021). Meanwhile, the flavonoids purified from Flammulina velutipes significantly increased the glutathione level, and the SOD activity of PC12 cells efficiently inhibited intracellular ROS accumulation (Hu et al., 2016). The phenolic compounds in the water extract of lentinus edobes and methanol extract of volvariella volvacea showed a higher hydrogen-providing ability to scavenge the DPPH radicals (Stajic et al., 2013).

### Proteins, terpenoids, arene, and lipids

Polysaccharides, polypeptides, and polyphenols are the major antioxidant compounds in mushrooms, while other compounds, including proteins, terpenes, arene, and lipids, tend to have antioxidant properties (Kuang et al., 2021). A novel protein from edible fungi could effectively remove OH and DPPH *in vitro*, while promoting the apoptosis of breast cancer cells through anti-tumor activity (Zhang et al., 2014). Terpenoids isolated and identified from Sanghuangporus sanghuang could scavenge DPPH and 2,2′-Azinobis-(3-ethylbenzthiazoline-6-sulphonate) (ABTS) free radicals; however, their antioxidant activity is significantly inferior to the polysaccharides and phenolic compounds (Zhang et al., 2021). P-terphenyl compounds isolated from mushroom Boletopsis leucomelas through chromatography possess effective DPPH scavenging capacity, which can be enhanced with heating (Sakemi et al., 2021). 2,5-diarylcyclopentenone derivatives from Paxillus involutus possess clearing abilities of DPPH,·OH, and O_2_—(Lv et al., 2021). Mushrooms are rich in fatty acids, much higher than in beef and pork, which might contribute to their antioxidant ability to fight against mitochondrial dysfunction (Fontes et al., 2019; Sande et al., 2019).

The antioxidant properties of compounds purified from mushrooms are affected by species, the extracted parts, and purification methods. Accumulating researchers have studied the polysaccharides, peptides, and phenolic compounds comprehensively, while studies focusing on protein, terpenoids, arene, and fatty acids are limited. The antioxidant effects of different compounds in mushrooms are presented in Table 1.

**TABLE 1:** Antioxidant effects of compounds purified from mushrooms.

Mushrooms	Compounds	Name	Antioxidant effects	References
*Lepista nuda*	Polysaccharide	LNP	Scavenge DPPH and O_2_ **·** ^**-**^	Shu et al. (2019)
*Entoloma lividoalbum*	Polysaccharide	ELPS	Eliminate **·**OH	Maity et al. (2015)
*Flammulina velutipes*	Polysaccharide	FVPs	Scavenge DPPH, **·**OH, and O_2_ **·** ^**-**^	Chen et al. (2019)
*Floral mushroom*	Polysaccharide	FMPS	Scavenge DPPH and **·**OH	Wang et al. (2015)
*Auricularia auricula*	Polysaccharide	AAP-3-1	Increase the activities of SOD, GSH-PX, and CAT	Qian et al. (2020)
*Oyster mushroom*	Polysaccharide	Extract	Improve the antioxidant status during ageing	Jayakumar et al. (2007)
*Pleurotus ostreatus*	Polysaccharide	Extract	Protect against oxidative damage induced by H_2_O_2_	Barbosa et al. (2020)
*Pleurotus djamor*	Polysaccharide	Extract	Scavenge DPPH and **·**OH	Maity et al. (2021)
*Pleurotus eryngii*	Polysaccharide	PERP	Scavenge reactive radicals and improve the antioxidant status	Zhang et al. (2021a)
*Hohenbuehelia serotina*	Polysaccharide	NTHSP-A1	Scavenging abilities of ABTS radical and **·**OH radical	Li et al. (2017b)
*Maitake*	Peptide	Glutathione	Antioxidant property	Kalaras et al. (2017)
*Matsutake*	Peptide	WFNNAGP	Scavenge **·**OH and promote the SOD activity	Li et al. (2021)
*Agaricus bisporus*	Peptide	MPI	Neutralize free radicals to resist oxidative stress	Kimatu et al. (2017)
*Schizophyllum commune*	Peptide	Extract	Free radical scavenging activity	Wongaem et al. (2021)
*Ophiocordyceps sinensis*	Peptide	COP	Scavenge DPPH radical and chelate heavy metal ions	Mishra et al. (2019)
*Hericium erinaceus*	Peptide	Extract	ABTS, DPPH and NO radical scavenging activities	Sangtitanu et al. (2020)
*Agaricus blazei*	Peptide	ABp	Change the contents of T-AOC, MDA, CAT, and ROS	Feng et al. (2021)
*Pleurotus eryngii*	Peptide	PEMP	Scavenge DPPH, **·**OH, and O_2_ **·** ^**-**^ radicals	Sun et al. (2017)
*Sanghuangporus sanghuang*	Polyphenol	Extract	Good cellular antioxidant activities	Zhang et al. (2021b)
*Flammulina velutipes*	Polyphenol	FFVP	Inhibit the secretion of NO and ROS	Ma et al. (2021)
*Phlebopus portentosus*	Polyphenol	Extract	DPPH scavenging activity and ferric reducing antioxidant power	Kumla et al. (2021)
*Phellinus linteus*	Polyphenol	Hispolon	Strong free radical scavenging ability	Sarfraz et al. (2020)
*Flammulina velutipes*	Polyphenol	FVF	Increase glutathione level and SOD activity and inhibit the accumulation of intracellular ROS	Hu et al. (2016)
*Boletus edulis* and *Cantharellus cibarius*	Polyphenol	Extract	The aqueous extract showed the strongest antioxidant activity	Fogarasi et al. (2021)
*Sanghuangporus baumii*	Polyphenol	Extract	Scavenge **·**OH, DPPH, and ABTS	Zheng et al. (2021)
*Boletopsis leucomelas*	P-terphenyl compound	Extract	Effective DPPH scavenging capacity	Sakemi et al. (2021)
*T. terrestris and T. vialis*	P-terphenyl compound	Extract	Prevent VEGF-induced production of ROS and malondialdehyde	Sonowal et al. (2018)
*Hericium erinaceum*	Sterol	Extract	Cellular antioxidant activity	Li et al. (2017a)
*Pholiota nameko*	Protein	PNAP	Scavenge **·**OH and DPPH	Zhang et al. (2014)
*Sanghuangporus sanghuang*	Terpenoid	Extract	Scavenge DPPH and ABTS free radicals	Zhang et al. (2021b)
*Paxillus involutus*	2,5-diarylcyclopentenone	Extract	Clearing abilities of DPPH, **·**OH, and O_2_ **·** ^**-**^	Lv et al. (2021)
*Agaricomycetes*	Extract	Extract	Significantly increase the activities of SOD, CAT and GSH-Px	Zhang et al. (2019)
*Agaricus bisporus*	Extract	Extract	Enhance the activities of antioxidant enzymes	Liu et al. (2013a)
*Lactarius salmonicolor*	Extract	Extract	Show the most potent radical scavenging activity	Athanasakis et al. (2013)
*Ramaria flava*	Extract	Extract	High DPPH and **·**OH radical-scavenging activities	Liu et al. (2013b)
*Chaga*	Extract	Extract	Scavenging activity against the ABTS radical cation and DPPH radical.	Lee et al. (2007)
*Porodaedalea chrysoloma*	Extract	Extract	Possess considerable antioxidant effect	Sarkozy et al. (2020)
*Orange coral mushroom*	Extract	Extract	Good free radical scavenges and reduce capacities	Aprotosoaie et al. (2017)
*Cynomorium coccineum*	Extract	Extract	ORAC-PYR assay gives the highest antioxidant value in both cases	Zucca et al. (2013)
*Entoloma lividoalbum*	Extract	Extract	Possess hydroxyl and superoxide radical-scavenging activities	Maity et al. (2014)
*Flammulina velutipes*	Extract	Extract	High DPPH radical scavenging activity	Bao et al. (2008)
*Pleurotus ostreatus*	Extract	Extract	High DPPH and hydrogen peroxide scavenging potential	Udeh et al. (2021)
*Agaricus brasiliensis*	Extract	Extract	Protect against sepsis by alleviating oxidative and inflammatory response	Navegantes-Lima et al. (2020)

## Applications of mushroom-derived compounds on chronic inflammatory diseases

### Cardiovascular disease

**FIGURE 4:**
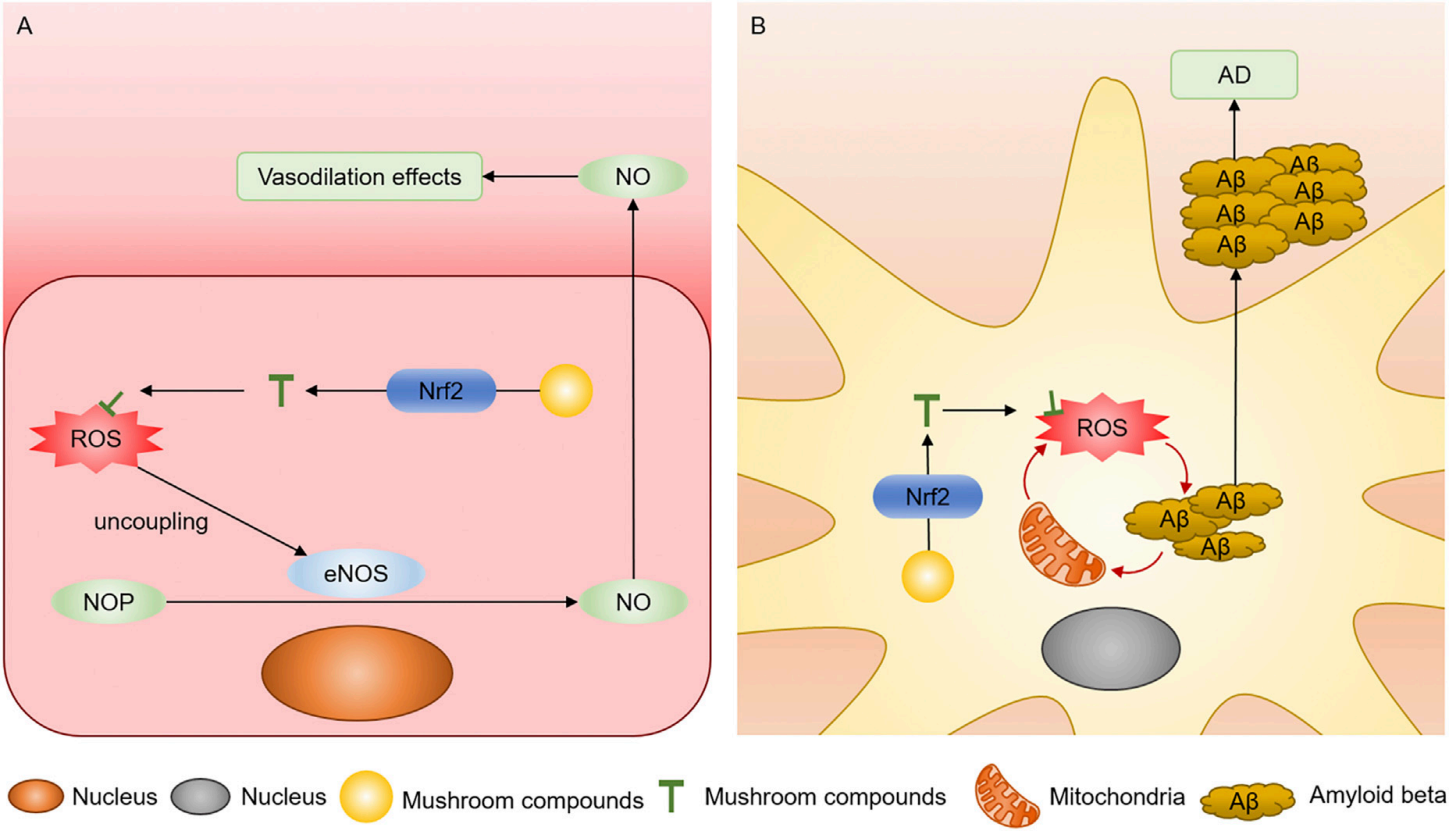
**(A)** Compounds in mushrooms inhibit eNOS uncoupling through the Nrf2 pathway. **(B)** Compounds in mushrooms neutralize ROS through the Nrf2 pathway and inhibit Aβ accumulation.

Endothelial cells act as the barrier between the blood and blood vessel wall, mediating the synthesis and release of a series of active substances (Miller, 2020). Oxidative stress associated with increased ROS production and reduced nitric oxide (NO) availability in the blood vessels and myocardium could induce endothelial dysfunction, including impaired vasodilation, proinflammatory, and thrombogenic, a risk factor for cardiovascular disease (Zhao et al., 2021). Endothelial-derived NO is the primary vasodilator and can react with increased ROS to form toxic peroxynitrite (ONOO−) (Meng et al., 2021). Meanwhile, ROS can induce the uncoupling of endothelial NO synthase (eNOS), a vital enzyme for NO production (Wu et al., 2014). The mushroom-derived compounds exert antioxidant effects through the Nrf2 pathway to effectively treat chronic cardiovascular diseases caused by endothelial disorders (Cheng et al., 2017; Shen et al., 2019) (Figure 4A).

Ergothioneine is highly abundant in mushrooms and has the ability to scour ROS and chelate the metal cations. This phenomenon of ergothioneine could effectively regulate endovascular inflammation caused by endothelial dysfunction and reduce the release of pro-inflammatory factors, including interleukin-6 (IL-6), IL-1β, and TNF-α (Lam-Sidun et al., 2021). The water-soluble polysaccharide FC isolated from wild mushroom agaricus bitorquis could inhibit the NADPH oxidase activity, which might be used to treat myocardial infarction, hypertension, and atherosclerosis caused by excessive ROS (Jiao et al., 2019). In a previous study, the vasodilatation of phellinus linteus extracts was found in the rat mesenteric artery, which might induce the antioxidant and anti-inflammatory effects of polysaccharides, peptides, and terpenoids in the mycelium (Cai et al., 2019; Kwon et al., 2020; Zuo et al., 2021).

### Neurodegenerative disease

Neurodegenerative diseases are characterized by the degeneration and loss of neurons in the brain and spinal cord, including Parkinson’s disease (PD), Alzheimer’s disease (AD), and Huntington’s disease (Djajadikerta et al., 2020). Lipids in the brain play a vital role in the function of neurons, which are vulnerable to being attacked by ROS and produce lipid peroxidation to form malondialdehyde (MDA), inducing reduced membrane fluidity and neuron degeneration (Singh et al., 2019). Meanwhile, the damaged neurons activate microglia and astrocytes to release the pro-inflammatory cytokines and ROS, further exacerbating neurodegeneration (Fischer and Maier, 2015). ROS produced by oxidative stress could damage and mutate the mitochondrial DNA, increasing oxidative decomposition of dopamine as the pathogenesis of neurodegenerative diseases and the accumulation of abnormal protein, such as amyloid beta (Aβ) in AD (Figure 4B) (Lin and Beal, 2006). Antioxidants in mushrooms could directly neutralize ROS or activate the Nrf2/ARE signaling pathway to induce the competence of antioxidant enzymes (Elfawy and Das, 2019).

Ethanol extracts from hericium erinaceus could alleviate the mitochondrial damage caused by H_2_O_2_ and activate antioxidant enzymes to treat hippocampal neurons’ injury in mice (Kushairi et al., 2019). The polysaccharide peptides and vitamins in coriolus versicolor could significantly reduce the proportion of GSH and oxidized glutathione (GSSG) in the plasma to relieve the systemic oxidative stress state and nerve injury of patients (Scuto et al., 2019). Meroterpenoids from Albatrellus yasudae have been found to inhibit Aβ aggregation, which might be a therapeutic and health care product for AD (Masuda et al., 2021). Polyphenols, polysaccharides, and triterpenes in Amauroderma rugosum could reduce oxidative stress and 6-hydroxydopamine-induced mitochondria dysfunction in the PC12 cells (Li et al., 2021b).

### Diabetes mellitus

The occurrence of diabetes mellitus is related to the dysfunction of islet *β* cells and insulin resistance, in which oxidative stress plays a vital role in regulating multiple signaling pathways (Zhang et al., 2020). Excessive ROS can activate the NF-κB, JNK/SAPK, and p38 MAPK pathways by activating TLR and inducing the dysfunction of islet β cells (Yaribeygi et al., 2020). A persistent hyperglycemic environment induces ROS production through the binding of advanced glycation end products (AGE) to peroxisome proliferators activated receptors (PPAR) (Deng et al., 2021). Additionally, excessive production of AGE increases the expression of inflammatory mediators through the NF-κB pathway (Karam et al., 2017). The inflammatory factor TNF-α activates the intracellular signaling factor IKKβ that affects insulin production through the NF-κB signaling pathway (Wellen and Hotamisligil, 2005; Rohm et al., 2022).

Polysaccharides isolated from Inonotus obliquus are the potential to treat type 2 diabetes by directly removing ROS or mediating lipid peroxidation (Lu et al., 2021). Terpenoids from Antrodia camphorata could significantly inhibit the binding of AGE to PPAR and reduce blood glucose levels, thus inhibiting ROS production and promoting insulin secretion (Kuang et al., 2021). Alkali-soluble polysaccharides from Amillariella mellea could improve pancreatic B-cell dysfunction caused by oxidative stress, thereby improving insulin sensitivity and reducing insulin resistance (Yang et al., 2019). As food supplements, *P. Ostreatus* and *L. Subnudus* have been shown to increase the activity of antioxidant enzymes and non-enzymatic antioxidants, inducing memory loss in diabetic rats (Agunloye and Oboh, 2021).

### Cancer

ROS can induce chronic inflammation, and the continuous inflammatory environment and oxidative stress might damage the adjacent epithelial cells and stromal cells, inducing cancer (Reuter et al., 2010). Additionally, ROS induces the proliferation of cancer cells and promotes tumor growth by activating the MAPK pathway (Moloney and Cotter, 2018). Tumor cell metastasis depends on the epithelial-mesenchymal transformation process, in which ROS activates the proteins, including β -catenin, e-cadherin, and matrix metalloproteinases (MMP), through the Wnt/β -catenin signaling pathway (Sosa et al., 2013).

Termitomyces Clypeatus significantly inhibited the tumor volume and number of ascites carcinoma mice by inhibiting lipid peroxidation and increasing the levels of GSH, SOD, and CAT (Mondal et al., 2016). The extracts from Lactarius deliciosus and Coprinus comatus inhibited the activity of MMP produced by oxidative stress and induced apoptosis in gliomas associated with G1 or G2/M phase cell cycle stagnation (Nowakowski et al., 2021). Natural antioxidants isolated from Thelephora Ganbajun by ultrasonic-assisted technology exhibited anti-proliferation effects on the liver and lung cancer cells (Xu et al., 2016). Antrodia salmonea induces protective autophagy and apoptosis in colon cancer cells through cascades of extracellular signal kinase (ERK) signaling, which has been reported to mediate ROS due to the double-sided effects of antioxidants on cancer cells (Dastmalchi et al., 2020; Yang et al., 2021).

The compounds purified from mushrooms might possess promising applications in preventing and treating cardiovascular diseases, neurodegenerative diseases, and cancer by regulating oxidative stress and chronic inflammation.

## Discussion

Excessive ROS increases the release of pro-inflammatory factors, thereby promoting ROS production, which is the primary reason for oxidative stress-induced chronic inflammation. Chronic inflammation can lead to organic diseases in different tissues and organs over time. Chronic inflammation in the blood vessels can affect the vascular endothelial relaxation function and form atherosclerosis, the root cause of coronary atherosclerotic heart disease (Golia et al., 2014). It is reported that chronic inflammatory response persists in the neurodegeneration of AD and is considered an important factor in accelerating the progression (Gasiorowski et al., 2018). In chronic inflammation, ROS can damage DNA and cause biological macromolecule dysfunction, such as proteins and lipids, inducing antioxidant dysfunction and a vicious cycle of oxidative stress, a risk factor for cancer (Murata, 2018). In recent years, antioxidants have attracted much attention due to their oxidative stress combating ability to inhibit chronic inflammation. Natural compounds are more easily recognized and absorbed by the body than synthetic antioxidants, with no toxic side effects (Liu, 2022). Chinese herbs, including Astragalus membranaceus, berberine, and curcumin have also been proven to have antioxidant properties (Kocaadam and Sanlier, 2017; Liu et al., 2017; Song et al., 2020). The separation and purification process of Chinese herbs extract is complicated, implying that purity can affect its application. Meanwhile, improper usage might lead to toxicoses (Hu et al., 2005; Liu et al., 2018). In contrast, mushrooms are widely distributed, easy to process, and can provide various nutrients with simple treatments (Ahmad et al., 2021). Additionally, mushrooms are well absorbed by the human body with no harm as the natural antioxidant (Fontes et al., 2019).

Mushrooms possess many biological and pharmacological characteristics, which are already applied in nutrition, health care, and medical treatment (Venturella et al., 2021). Therefore, studies pay more attention to mushroom-derived polysaccharides than other compounds, as the purification method is mature and the biological activity is stable and diverse (Muszynska et al., 2018). Mushroom polysaccharides mainly contain β-glucan chains, which have been proven to have antioxidant effects (Maity et al., 2021). The hydrogen ions of mushroom polysaccharides could inhibit oxidative stress by neutralizing ROS and enhance antioxidant effects through chemical modification, including carboxymethylation and sulphuration (Huang and Nie, 2015; Lu et al., 2020). The extraction method significantly affected the antioxidant activity of the compound. Water extraction of Lepista nuda polysaccharides showed better scavenging ability of free radicals than ethanol extraction (Shu et al., 2019). The same compound had different scavenging abilities for different free radicals. For instance, Sanghuang could scavenge 68% ·OH and 57% O_2_—at 1 mg/ml, indicating that the scavenging ability of most compounds in the mushrooms is concentration-dependent (Zuo et al., 2021). Mushrooms are a promising source of natural antioxidants, ergothioneine, and glutathione, with the highest content in the mushroom cap of yellow oyster (Kalaras et al., 2017). However, more research on different species of natural compounds purified from mushrooms should be conducted to broaden their application.

In conclusion, the compounds in mushrooms could inhibit oxidative stress and chronic inflammation by neutralizing excessive ROS or activating antioxidant enzymes through the Nrf2 signaling pathway and have been applied to cardiovascular disease, neurodegenerative disease, and cancer.

## Supplementary material

SUPPLEMENTARY FIGURE S1
Structure of compounds purified from mushrooms, with hydrogen atoms in red as active sites against oxidative stress. N is the number of cycles. **(A)** Structure of mushroom polysaccharide. **(B)** Structure of mushroom peptides. **(C)** Structure of mushroom polyphenols, with flavonoids as an example.
