# Corrigendum: Compounds purified from edible fungi fight against chronic inflammation through oxidative stress regulation

**DOI:** 10.3389/fphar.2022.1081523

**Published:** 2023-01-05

**Authors:** Yidan Xia, Dongxu Wang, Jiaqi Li, Minqi Chen, Duo Wang, Ziping Jiang, Bin Liu

**Affiliations:** ^1^ Department of Hand and Foot Surgery, The First Hospital of Jilin University, Changchun, China; ^2^ Laboratory Animal Center, College of Animal Science, Jilin University, Changchun, China

**Keywords:** chronic diseases, natural compounds, edible fungi, antioxidants, molecular mechanisms

In the published article, the reference for “Recently, various compounds have been isolated from mushrooms, such as polysaccharides, alkaloids, peptides, terpenoids, and polyphenols (Leong et al., 2021)” was incorrectly written as (Leong et al., 2021). It should be (Homer and Sperry, 2017; Zhou et al., 2020; Kuang et al., 2021; Leong et al., 2021; Zhang et al., 2021).

In the published article, there was an error in Table 1 as published. The references of Table 1 were incorrect due to our carelessness in proof section. The corrected Table 1 and its caption (Table 1 Antioxidant effects of compounds purified from mushrooms) appear below.

**TABLE 1:** Antioxidant effects of compounds purified from mushrooms.

Mushrooms	Compounds	Name	Antioxidant effects	References
*Lepista nuda*	Polysaccharide	LNP	Scavenge DPPH and O_2_ **·** ^**-**^	Shu et al. (2019)
*Entoloma lividoalbum*	Polysaccharide	ELPS	Eliminate **·**OH	Maity et al. (2015)
*Flammulina velutipes*	Polysaccharide	FVPs	Scavenge DPPH, **·**OH, and O_2_ **·** ^**-**^	Chen et al. (2019)
*Floral mushroom*	Polysaccharide	FMPS	Scavenge DPPH and **·**OH	Wang et al. (2015)
*Auricularia auricula*	Polysaccharide	AAP-3-1	Increase the activities of SOD, GSH-PX, and CAT	Qian et al. (2020)
*Oyster mushroom*	Polysaccharide	Extract	Improve the antioxidant status during ageing	Jayakumar et al. (2007)
*Pleurotus ostreatus*	Polysaccharide	Extract	Protect against oxidative damage induced by H_2_O_2_	Barbosa et al. (2020)
*Pleurotus djamor*	Polysaccharide	Extract	Scavenge DPPH and **·**OH	Maity et al. (2021)
*Pleurotus eryngii*	Polysaccharide	PERP	Scavenge reactive radicals and improve the antioxidant status	Zhang et al. (2021a)
*Hohenbuehelia serotina*	Polysaccharide	NTHSP-A1	Scavenging abilities of ABTS radical and **·**OH radical	Li et al. (2017b)
*Maitake*	Peptide	Glutathione	Antioxidant property	Kalaras et al. (2017)
*Matsutake*	Peptide	WFNNAGP	Scavenge **·**OH and promote the SOD activity	Li et al. (2021)
*Agaricus bisporus*	Peptide	MPI	Neutralize free radicals to resist oxidative stress	Kimatu et al. (2017)
*Schizophyllum commune*	Peptide	Extract	Free radical scavenging activity	Wongaem et al. (2021)
*Ophiocordyceps sinensis*	Peptide	COP	Scavenge DPPH radical and chelate heavy metal ions	Mishra et al. (2019)
*Hericium erinaceus*	Peptide	Extract	ABTS, DPPH and NO radical scavenging activities	Sangtitanu et al. (2020)
*Agaricus blazei*	Peptide	ABp	Change the contents of T-AOC, MDA, CAT, and ROS	Feng et al. (2021)
*Pleurotus eryngii*	Peptide	PEMP	Scavenge DPPH, **·**OH, and O_2_ **·** ^**-**^ radicals	Sun et al. (2017)
*Sanghuangporus sanghuang*	Polyphenol	Extract	Good cellular antioxidant activities	Zhang et al. (2021b)
*Flammulina velutipes*	Polyphenol	FFVP	Inhibit the secretion of NO and ROS	Ma et al. (2021)
*Phlebopus portentosus*	Polyphenol	Extract	DPPH scavenging activity and ferric reducing antioxidant power	Kumla et al. (2021)
*Phellinus linteus*	Polyphenol	Hispolon	Strong free radical scavenging ability	Sarfraz et al. (2020)
*Flammulina velutipes*	Polyphenol	FVF	Increase glutathione level and SOD activity and inhibit the accumulation of intracellular ROS	Hu et al. (2016)
*Boletus edulis* and *Cantharellus cibarius*	Polyphenol	Extract	The aqueous extract showed the strongest antioxidant activity	Fogarasi et al. (2021)
*Sanghuangporus baumii*	Polyphenol	Extract	Scavenge **·**OH, DPPH, and ABTS	Zheng et al. (2021)
*Boletopsis leucomelas*	P-terphenyl compound	Extract	Effective DPPH scavenging capacity	Sakemi et al. (2021)
*T. terrestris and T. vialis*	P-terphenyl compound	Extract	Prevent VEGF-induced production of ROS and malondialdehyde	Sonowal et al. (2018)
*Hericium erinaceum*	Sterol	Extract	Cellular antioxidant activity	Li et al. (2017a)
*Pholiota nameko*	Protein	PNAP	Scavenge **·**OH and DPPH	Zhang et al. (2014)
*Sanghuangporus sanghuang*	Terpenoid	Extract	Scavenge DPPH and ABTS free radicals	Zhang et al. (2021b)
*Paxillus involutus*	2,5-diarylcyclopentenone	Extract	Clearing abilities of DPPH, **·**OH, and O_2_ **·** ^**-**^	Lv et al. (2021)
*Agaricomycetes*	Extract	Extract	Significantly increase the activities of SOD, CAT and GSH-Px	Zhang et al. (2019)
*Agaricus bisporus*	Extract	Extract	Enhance the activities of antioxidant enzymes	Liu et al. (2013a)
*Lactarius salmonicolor*	Extract	Extract	Show the most potent radical scavenging activity	Athanasakis et al. (2013)
*Ramaria flava*	Extract	Extract	High DPPH and **·**OH radical-scavenging activities	Liu et al. (2013b)
*Chaga*	Extract	Extract	Scavenging activity against the ABTS radical cation and DPPH radical.	Lee et al. (2007)
*Porodaedalea chrysoloma*	Extract	Extract	Possess considerable antioxidant effect	Sarkozy et al. (2020)
*Orange coral mushroom*	Extract	Extract	Good free radical scavenges and reduce capacities	Aprotosoaie et al. (2017)
*Cynomorium coccineum*	Extract	Extract	ORAC-PYR assay gives the highest antioxidant value in both cases	Zucca et al. (2013)
*Entoloma lividoalbum*	Extract	Extract	Possess hydroxyl and superoxide radical-scavenging activities	Maity et al. (2014)
*Flammulina velutipes*	Extract	Extract	High DPPH radical scavenging activity	Bao et al. (2008)
*Pleurotus ostreatus*	Extract	Extract	High DPPH and hydrogen peroxide scavenging potential	Udeh et al. (2021)
*Agaricus brasiliensis*	Extract	Extract	Protect against sepsis by alleviating oxidative and inflammatory response	Navegantes-Lima et al. (2020)

The authors apologize for this error and state that this does not change the scientific conclusions of the article in any way. The original article has been updated.
