# Supplementary material for: Compounds purified from edible fungi fight against chronic inflammation through oxidative stress regulation
Source: Front Pharmacol. 2022 Sep 9;13:974794. doi: 10.3389/fphar.2022.974794 (PMC9500316; doi:10.3389/fphar.2022.974794)
Supplement: Supplementary file 1 [file Table1.DOCX]

**Table.1** Antioxidant effects of compounds purified from mushrooms.

| **Mushrooms** | **Compounds** | **Name** | **Antioxidant effects** | **Ref** |
| --- | --- | --- | --- | --- |
| *Lepista nuda* | Polysaccharide | LNP | Scavenge DPPH and O_2_**·^-^** | [1] |
| *Entoloma lividoalbum* | Polysaccharide | ELPS | Eliminate **·**OH | [2] |
| *Flammulina velutipes* | Polysaccharide | FVPs | Scavenge DPPH, **·**OH, and O_2_**·^-^** | [3] |
| *Floral mushroom* | Polysaccharide | FMPS | Scavenge DPPH and **·**OH | [4] |
| *Auricularia auricula* | Polysaccharide | AAP-3-1 | Increase the activities of SOD, GSH-PX, and CAT | [5] |
| *Oyster mushroom* | Polysaccharide | Extract | Improve the antioxidant status during ageing | [6] |
| *Pleurotus ostreatus* | Polysaccharide | Extract | Protect against oxidative damage induced by H_2_O_2_ | [7] |
| *Pleurotus djamor* | Polysaccharide | Extract | Scavenge DPPH and **·**OH | [8] |
| *Pleurotus eryngii* | Polysaccharide | PERP | Scavenge reactive radicals and improve the antioxidant status | [9] |
| *Hohenbuehelia serotina* | Polysaccharide | NTHSP-A1 | Scavenging abilities of ABTS radical and **·**OH radical | [10] |
| *Maitake* | Peptide | Glutathione | Antioxidant property | [11] |
| *Matsutake* | Peptide | WFNNAGP | Scavenge **·**OH and promote the SOD activity | [12] |
| *Agaricus bisporus* | Peptide | MPI | Neutralize free radicals to resist oxidative stress | [13] |
| *Chizophyllum commune* | Peptide | Extract | Concentration-dependent free radical scavenging activity | [14] |
| *Ophiocordyceps sinensis* | Peptide | COP | Scavenge DPPH radical and chelate heavy metal ions | [15] |
| *Hericium erinaceus* | Peptide | Extract | ABTS, DPPH and NO radical scavenging activities | [16] |
| *Agaricus blazei* | Peptide | ABp | Change the contents of T-AOC, MDA, CAT, and ROS | [17] |
| *Pleurotus eryngii* | Peptide | PEMP | Scavenge DPPH, **·**OH, and O_2_**·^-^** radicals | [18] |
| *Sanghuangporus sanghuang* | Polyphenol | Extract | Good cellular antioxidant activities | [19] |
| *Flammulina velutipes* | Polyphenol | FFVP | Inhibit the secretion of NO and ROS | [20] |
| *Phlebopus portentosus* | Polyphenol | Extract | DPPH scavenging activity and ferric reducing antioxidant power | [21] |
| *Phellinus linteus* | Polyphenol | Hispolon | Strong free radical scavenging ability | [22] |
| *Flammulina velutipes* | Polyphenol | FVF | Increase glutathione level and SOD activity and inhibit the accumulation of intracellular ROS | [23] |
| *Boletus edulis* and *Cantharellus cibarius* | Polyphenol | Extract | The aqueous extract showed the strongest antioxidant activity | [24] |
| *Sanghuangporus baumii* | Polyphenol | Extract | Scavenge **·**OH, DPPH, and ABTS | [25] |
| *Boletopsis leucomelas* | P-terphenyl compound | Extract | Effective DPPH scavenging capacity | [26] |
| *T. terrestris and T. vialis* | P-terphenyl compound | Extract | Prevent VEGF-induced production of ROS and malondialdehyde | [27] |
| *Hericium erinaceum* | Sterol | Extract | Cellular antioxidant activity | [28] |
| *Pholiota nameko* | Protein | PNAP | Scavenge **·**OH and DPPH | [29] |
| *Sanghuangporus sanghuang* | Terpenoid | Extract | Scavenge DPPH and ABTS free radicals | [19] |
| *Paxillus involutus* | 2,5-diarylcyclopentenone | Extract | Clearing abilities of DPPH, **·**OH, and O_2_**·^-^** | [30] |
| *Agaricomycetes* | Extract | Extract | Significantly increase the activities of SOD, CAT and GSH-Px | [31] |
| *Agaricus bisporus* | Extract | Extract | Enhance the activities of antioxidant enzymes | [32] |
| *Lactarius salnicolor* | Extract | Extract | Show the most potent radical scavenging activity | [33] |
| *Ramaria flava* | Extract | Extract | High DPPH and **·**OH radical-scavenging activities | [34] |
| *Chaga* | Extract | Extract | Scavenging activity against the ABTS radical cation and DPPH radical. | [35] |
| *Porodaedalea chrysoloma* | Extract | Extract | Possess considerable antioxidant effect | [36] |
| *Orange coral mushroom* | Extract | Extract | Good free radical scavenges and reduce capacities | [37] |
| *Cynomorium coccineum* | Extract | Extract | ORAC-PYR assay gives the highest antioxidant value in both cases | [38] |
| *Entoloma lividoalbum* | Extract | Extract | Possess hydroxyl and superoxide radical-scavenging activities | [39] |
| *Flammulina velutipes* | Extract | Extract | High DPPH radical scavenging activity | [40] |
| *Pleurotus ostreatus* | Extract | Extract | High DPPH and hydrogen peroxide scavenging potential | [41] |
| *Agaricus brasiliensis* | Extract | Extract | Protect against sepsis by alleviating oxidative and inflammatory response | [42] |

Reference:

1. Shu, X., et al., *Extraction, purification and properties of water-soluble polysaccharides from mushroom Lepista nuda.* Int J Biol Macromol, 2019. **128**: p. 858-869.

2. Maity, P., et al., *Structural, immunological, and antioxidant studies of beta-glucan from edible mushroom Entoloma lividoalbum.* Carbohydr Polym, 2015. **123**: p. 350-8.

3. Chen, X., et al., *Effects of ultrasound-assisted extraction on antioxidant activity and bidirectional immunomodulatory activity of Flammulina velutipes polysaccharide.* Int J Biol Macromol, 2019. **140**: p. 505-514.

4. Wang, J.H., et al., *Physicochemical properties and antioxidant activities of polysaccharide from floral mushroom cultivated in Huangshan Mountain.* Carbohydr Polym, 2015. **131**: p. 240-7.

5. Qian, L., et al., *Purification, characterization and in vitro antioxidant activity of a polysaccharide AAP-3-1 from Auricularia auricula.* Int J Biol Macromol, 2020. **162**: p. 1453-1464.

6. Jayakumar, T., P.A. Thomas, and P. Geraldine, *Protective effect of an extract of the oyster mushroom, Pleurotus ostreatus, on antioxidants of major organs of aged rats.* Exp Gerontol, 2007. **42**(3): p. 183-91.

7. Barbosa, J.R., et al., *Obtaining extracts rich in antioxidant polysaccharides from the edible mushroom Pleurotus ostreatus using binary system with hot water and supercritical CO2.* Food Chem, 2020. **330**: p. 127173.

8. Maity, G.N., et al., *Structural features and antioxidant activity of a new galactoglucan from edible mushroom Pleurotus djamor.* Int J Biol Macromol, 2021. **168**: p. 743-749.

9. Zhang, C., et al., *Antioxidant and anti-ageing effects of enzymatic polysaccharide from Pleurotus eryngii residue.* Int J Biol Macromol, 2021. **173**: p. 341-350.

10. Li, X., L. Wang, and Z. Wang, *Structural characterization and antioxidant activity of polysaccharide from Hohenbuehelia serotina.* Int J Biol Macromol, 2017. **98**: p. 59-66.

11. Kalaras, M.D., et al., *Mushrooms: A rich source of the antioxidants ergothioneine and glutathione.* Food Chem, 2017. **233**: p. 429-433.

12. Li, M., et al., *Tricholoma matsutake-derived peptide WFNNAGP protects against DSS-induced colitis by ameliorating oxidative stress and intestinal barrier dysfunction.* Food Funct, 2021. **12**(23): p. 11883-11897.

13. Kimatu, B.M., et al., *Antioxidant potential of edible mushroom (Agaricus bisporus) protein hydrolysates and their ultrafiltration fractions.* Food Chem, 2017. **230**: p. 58-67.

14. Wongaem, A., et al., *Antioxidant properties of peptides obtained from the split gill mushroom (Schizophyllum commune).* J Food Sci Technol, 2021. **58**(2): p. 680-691.

15. Mishra, J., et al., *Antioxidant-Rich Peptide Fractions Derived from High-Altitude Chinese Caterpillar Medicinal Mushroom Ophiocordyceps sinensis (Ascomycetes) Inhibit Bacterial Pathogens.* Int J Med Mushrooms, 2019. **21**(2): p. 155-168.

16. Sangtitanu, T., et al., *Peptides obtained from edible mushrooms: Hericium erinaceus offers the ability to scavenge free radicals and induce apoptosis in lung cancer cells in humans.* Food Funct, 2020. **11**(6): p. 4927-4939.

17. Feng, Q., et al., *Agaricus blazei polypeptide exerts a protective effect on D-galactose-induced aging mice via the Keap1/Nrf2/ARE and P53/Trim32 signaling pathways.* J Food Biochem, 2021. **45**(1): p. e13555.

18. Sun, Y., X. Hu, and W. Li, *Antioxidant, antitumor and immunostimulatory activities of the polypeptide from Pleurotus eryngii mycelium.* Int J Biol Macromol, 2017. **97**: p. 323-330.

19. Zhang, J.J., et al., *Sesquiterpenes and polyphenols with glucose-uptake stimulatory and antioxidant activities from the medicinal mushroom Sanghuangporus sanghuang.* Chin J Nat Med, 2021. **19**(9): p. 693-699.

20. Ma, S., H. Zhang, and J. Xu, *Characterization, Antioxidant and Anti-Inflammation Capacities of Fermented Flammulina velutipes Polyphenols.* Molecules, 2021. **26**(20).

21. Kumla, J., et al., *Comparative Evaluation of Chemical Composition, Phenolic Compounds, and Antioxidant and Antimicrobial Activities of Tropical Black Bolete Mushroom Using Different Preservation Methods.* Foods, 2021. **10**(4).

22. Sarfraz, A., et al., *Hispolon: A natural polyphenol and emerging cancer killer by multiple cellular signaling pathways.* Environ Res, 2020. **190**: p. 110017.

23. Hu, Q., et al., *Identification of flavonoids from Flammulina velutipes and its neuroprotective effect on pheochromocytoma-12 cells.* Food Chem, 2016. **204**: p. 274-282.

24. Fogarasi, M., et al., *Comparison of Different Extraction Solvents for Characterization of Antioxidant Potential and Polyphenolic Composition in Boletus edulis and Cantharellus cibarius Mushrooms from Romania.* Molecules, 2021. **26**(24).

25. Zheng, N., et al., *Optimization of Extraction Process and the Antioxidant Activity of Phenolics from Sanghuangporus baumii.* Molecules, 2021. **26**(13).

26. Sakemi, Y., et al., *Antioxidant p-terphenyl compounds in the mushroom Boletopsis leucomelas (PERS.) FAYOD and how they change via cooking.* Food Chem, 2021. **363**: p. 130281.

27. Sonowal, H., et al., *Vialinin A, an Edible Mushroom-Derived p-Terphenyl Antioxidant, Prevents VEGF-Induced Neovascularization In Vitro and In Vivo.* Oxid Med Cell Longev, 2018. **2018**: p. 1052102.

28. Li, W., et al., *Antioxidant and Anti-Osteoporotic Activities of Aromatic Compounds and Sterols from Hericium erinaceum.* Molecules, 2017. **22**(1).

29. Zhang, Y., et al., *Purification and characterization of a novel antitumor protein with antioxidant and deoxyribonuclease activity from edible mushroom Pholiota nameko.* Biochimie, 2014. **99**: p. 28-37.

30. Lv, J.H., et al., *Novel 2,5-Diarylcyclopentenone Derivatives from the Wild Edible Mushroom Paxillus involutus and Their Antioxidant Activities.* J Agric Food Chem, 2021. **69**(17): p. 5040-5048.

31. Zhang, J., et al., *Antioxidant and Anti-Aging Activities of Ethyl Acetate Extract of the Coral Tooth Mushroom, Hericium coralloides (Agaricomycetes).* Int J Med Mushrooms, 2019. **21**(6): p. 561-570.

32. Liu, J., et al., *In vitro and in vivo antioxidant activity of ethanolic extract of white button mushroom (Agaricus bisporus).* Food Chem Toxicol, 2013. **51**: p. 310-6.

33. Athanasakis, G., et al., *Antioxidant properties of the wild edible mushroom Lactarius salmonicolor.* J Med Food, 2013. **16**(8): p. 760-4.

34. Liu, K., et al., *Anticancer, antioxidant and antibiotic activities of mushroom Ramaria flava.* Food Chem Toxicol, 2013. **58**: p. 375-80.

35. Lee, I.K., et al., *New antioxidant polyphenols from the medicinal mushroom Inonotus obliquus.* Bioorg Med Chem Lett, 2007. **17**(24): p. 6678-81.

36. Sarkozy, A., et al., *Isolation and Characterization of Chemical Constituents from the Poroid Medicinal Mushroom Porodaedalea chrysoloma (Agaricomycetes) and Their Antioxidant Activity.* Int J Med Mushrooms, 2020. **22**(2): p. 125-131.

37. Aprotosoaie, A.C., et al., *Antioxidant and antigenotoxic potential of Ramaria largentii Marr & D. E. Stuntz, a wild edible mushroom collected from Northeast Romania.* Food Chem Toxicol, 2017. **108**(Pt B): p. 429-437.

38. Zucca, P., et al., *Evaluation of antioxidant potential of "maltese mushroom" (Cynomorium coccineum) by means of multiple chemical and biological assays.* Nutrients, 2013. **5**(1): p. 149-61.

39. Maity, P., et al., *Structure elucidation and antioxidant properties of a soluble beta-D-glucan from mushroom Entoloma lividoalbum.* Int J Biol Macromol, 2014. **63**: p. 140-9.

40. Bao, H.N., H. Ushio, and T. Ohshima, *Antioxidative activity and antidiscoloration efficacy of ergothioneine in mushroom (Flammulina velutipes) extract added to beef and fish meats.* J Agric Food Chem, 2008. **56**(21): p. 10032-40.

41. Udeh, A.S., et al., *Antibacterial and Antioxidant Activity of Different Extracts of Some Wild Medicinal Mushrooms from Nigeria.* Int J Med Mushrooms, 2021. **23**(10): p. 83-95.

42. Navegantes-Lima, K.C., et al., *Agaricus brasiliensis Mushroom Protects Against Sepsis by Alleviating Oxidative and Inflammatory Response.* Front Immunol, 2020. **11**: p. 1238.
