# Supplementary figures and images for: Compounds purified from edible fungi fight against chronic inflammation through oxidative stress regulation
Source: Front Pharmacol. 2022 Sep 9;13:974794. doi: 10.3389/fphar.2022.974794 (PMC9500316; doi:10.3389/fphar.2022.974794)

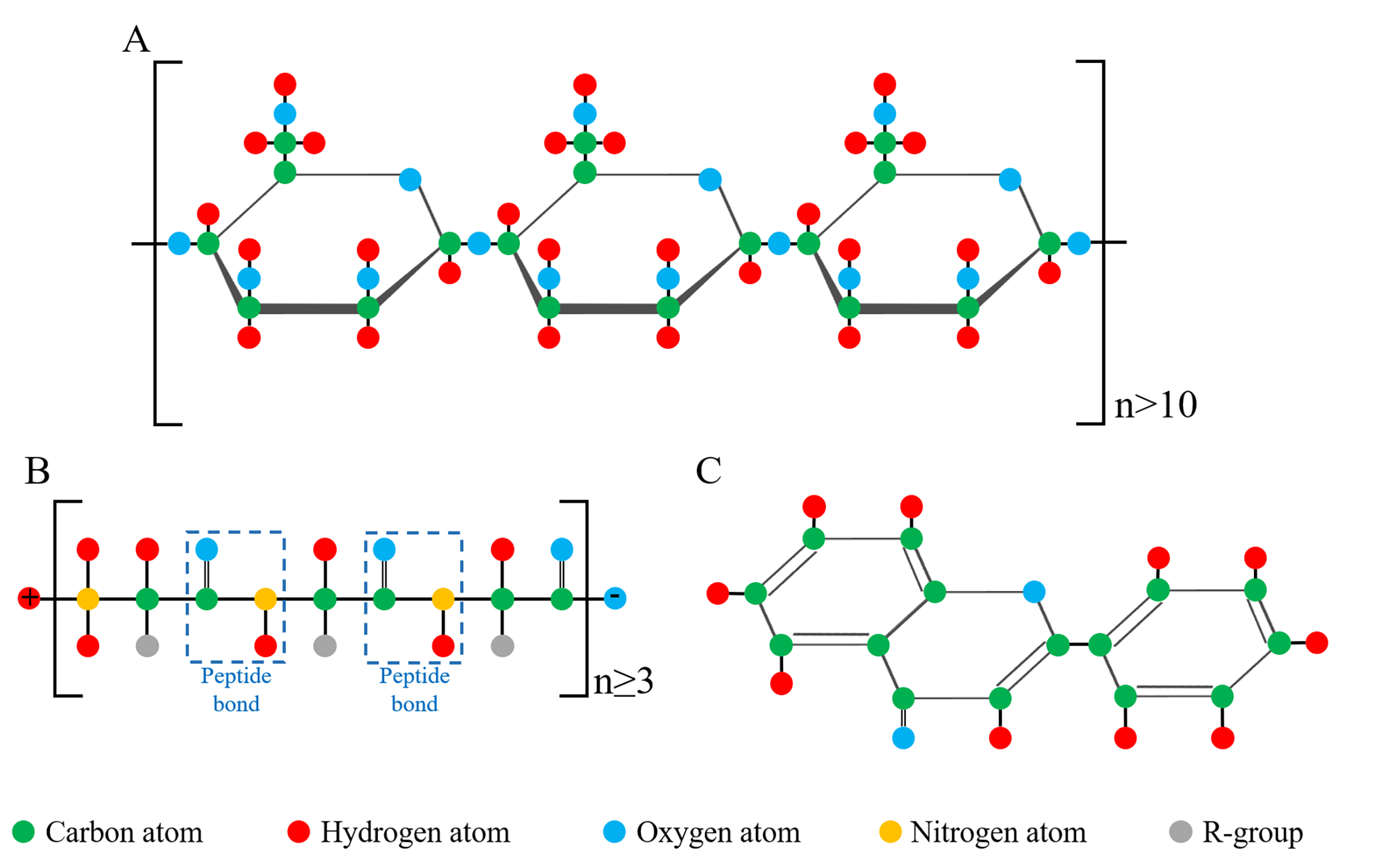

Supplement: Supplementary file 2 [file Image1.TIF]
